# Development and pilot evaluation of an evidence-based algorithm for MASLD (formerly NAFLD) management in primary care in Europe

**DOI:** 10.3389/fmed.2024.1383112

**Published:** 2024-11-21

**Authors:** Marilena Anastasaki, Sophia Papadakis, Irini N. Gergianaki, Loucas Papastamatiou, Eftychios Aligizakis, Nikoleta Grillaki, Eleni Boutzoukaki, Nektarios Sivaropoulos, Foteini Anastasiou, Juan Mendive, Carlos de Juan-Asenjo, Rosario Hernández-Ibáñez, Alba Martínez-Escudé, Montserrat Garcia-Retortillo, Ger Koek, Leen Heyens, Jean Muris, Christos D. Lionis

**Affiliations:** ^1^Clinic of Social and Family Medicine, School of Medicine, University of Crete, Heraklion, Greece; ^2^Kandanos Healthcare Center, Chania, Greece; ^3^Second Healthcare Center of Chania, Chania, Greece; ^4^Alikianos Rural Practice, Chania, Greece; ^5^Spili Healthcare Center, Rethymno, Greece; ^6^European Society for Primary Care Gastroenterology, Stockholm, Sweden; ^7^La Mina Primary Health Care Centre—IDIAP Jordi Gol, Barcelona, Spain; ^8^La Marina Health Centre, Barcelona, Spain; ^9^MASLD Working Group, Catalan Society of Family Medicine (CAMFiC), Barcelona, Spain; ^10^La LLagosta Primary Health Care Centre, La Llagosta, Barcelona, Spain; ^11^Hepatology Unit, Hospital del Mar, Barcelona, Spain; ^12^Department of Gastroenterology and Hepatology, Maastricht University Medical Center, Maastricht, Netherlands; ^13^Faculty of Health and Life Sciences, Hasselt University, Diepenbeek, Belgium; ^14^School of Nutrition and Translational Research in Metabolism, NUTRIM, Maastricht University, Maastricht, Netherlands; ^15^Department of Endocrinology, Ziekenhuis Oost-Limburg, Genk, Belgium; ^16^Department of Family Medicine, CAPHRI Research Institute, Maastricht University, Maastricht, Netherlands

**Keywords:** metabolic dysfunction-associated steatotic liver disease (MASLD), primary care, clinical care pathways, risk classification, non-alcoholic fatty liver disease (NAFLD), screening, management

## Abstract

**Background:**

Metabolic dysfunction-associated steatotic liver disease (MASLD, formerly NAFLD), emerges as major cause of morbidity and mortality globally, with chronic patients facing increased risk. Guidelines on MASLD management in primary care (PC) are limited. This study aimed to develop and evaluate a clinical care pathway for use in PC to improve MASLD screening and management, including early detection, communication and treatment, in three European countries (Greece, Spain, the Netherlands).

**Methods:**

An international multidisciplinary panel of experts oversaw pathway development, which was designed as a two-step algorithm with defined and sequenced tasks. To evaluate algorithm implementation, a controlled pilot study was conducted. Patients at risk of MASLD were assigned to general practitioners (GPs) trained in algorithm implementation (active group) or usual care (control group) and followed for 4–8 weeks. Primary outcomes were the number of patients screened for MASLD, managed in PC and referred to specialists.

**Results:**

In this algorithm, patients with metabolic or liver dysfunction, confirmed MASLD or cardiovascular disease are screened with FIB-4 and classified as having risk of low-level (FIB-4 < 1.3), intermediate-level (1.3 ≤ FIB-4 < 2.67) or high-level MASLD (FIB-4 ≥ 2.67). The algorithm provides evidence-based tools to support GPs manage patients with risk of low-level MASLD in PC, coordinate linkage of patients with risk of high-level MASLD to specialists and refer patients with risk of intermediate-level MASLD for elastography (low-risk if <7.9 kPa or intermediate/high-risk if ≥7.9 kPa). During pilot evaluation, *N* = 37 participants were recruited in Spain (54.1% women, median age: 63 years). Significantly higher rates of patients in the active group (*n* = 17) than the control group (*n* = 20) were screened with FIB-4 (94.1% vs. 5.5%, *p* = 0.004). Patients in the active group received significantly more frequently a PC intervention for weight loss (70.6% vs. 10.0%, *p* < 0.001), alcohol regulation (52.9% vs. 0%, *p* < 0.001) and smoking cessation (29.4% vs. 0%, *p* = 0.005). In Greece no algorithm implementation was observed in either the active or control group, while the evaluation was not conducted in the Netherlands for logistic reasons.

**Conclusion:**

This study provides evidence on the development and implementation of a new PC algorithm for MASLD screening and management. Variations among participating settings in algorithm implementation are indicative of context-specific particularities. Further research is necessary for integrating such pathways in tailored interventions to tackle this emerging public health issue.

## Introduction

Metabolic dysfunction-associated steatotic liver disease (MASLD), formerly non-alcoholic fatty liver disease (NAFLD) is the most common liver disorder. Metabolic dysfunction-associated steatohepatitis (MASH), formerly non-alcoholic steatohepatitis (NASH) is its most aggressive manifestation and is characterised by cell damage and inflammation which can further progress to fibrosis, cirrhosis and hepatocellular carcinoma (1). It is expected that MASH will become the leading cause of liver transplantation within the next years (2), while it is currently the main risk factor of hepatocellular carcinoma (3). Despite the significant burden on public health, appropriate suspicion, screening, identification, and linkage to care of patients with signs of advanced fibrosis remain an unmet need.

Since obesity, metabolic syndrome and diabetes are the most frequent co-morbidities in chronic liver disease, patients at high risk for MASLD are often managed in primary care (PC) and followed up by general practitioners (GPs). Although liver fibrosis staging is critical for diagnosing MASLD (4, 5), it is difficult to identify patients with significant fibrosis in primary care due to limited access to fibrosis tests. Without comprehensive guidance and awareness, proper referral to specialty care for high-risk patients is also challenging for GPs (6). Patients with mild disease are often referred when the appropriate preventative interventions of lifestyle changes can be delivered effectively in PC (7). In contrast, advanced fibrosis or cirrhosis is often under-estimated, remaining undetected and leading to late diagnosis of progressed disease. In the absence of comprehensive pharmacological treatment for advanced fibrosis (8), the use of readily available non-invasive tests, standardized referral and treatment algorithms, as well as multi-disciplinary collaboration between GP, endocrinology, diabetology, hepatology, cardiovascular and obesity specialists are key factors for optimal care delivery.

Evidence on non-invasive liver fibrosis tests and innovative pathways for the earlier identification of patients with chronic liver disease and subsequent access to specialist care indicates promising results. A study evaluating a clinical care pathway for patients identified with MASLD using non-invasive fibrosis assessment to stratify patients suggested that the pathway detected five times more cases of advanced fibrosis and cirrhosis while reducing unnecessary referrals from primary to secondary care by 81% (9). Still, comprehensive guidance on such diagnostics and processes remains needed (10), while there is little development and evaluation of similar clinical care pathways for MASLD in PC internationally (11). Such pathways can help address bottlenecks and can be used as part of a comprehensive action plan for screening individuals at risk and providing appropriate referral, intervention, and follow-up. Evaluation and validation of such models is, however, necessary for establishing their effectiveness, including factors related to process, outcomes and feasibility and guide necessary adjustments for achieving optimal adaptation, impact and integration.

The overall aim of this study was to develop an evidence-based pathway to enhance the screening and management of MASLD in primary care, including detection, communication and treatment. We also sought to adapt the pathway to the local cultural and clinical practice contexts of three European countries with diverse health care systems (Greece, Spain, the Netherlands) and evaluate its implementation in a pilot observational study.

## Methods

### Pathway development

#### Design

The pathway was designed as a standardized clinical care algorithm with defined, optimized and sequenced tasks developed through an expert panel consensus.

#### Target population

The algorithm was designed for use by GPs in Greece, Spain and the Netherlands.

#### Outcomes

The primary outcome of this activity was the documentation of the MASLD algorithm in terms of best practices, guidelines, theoretical framework, patient journey, care pathway, barriers/solutions, quality improvement, implementation procedures/tools and evaluation tools. A secondary outcome was the documentation of local adaptations performed per model domain in each country.

#### Theoretical framework

The Chronic Care Model was used to guide pathway development as it provides the background to shift from acute, episodic and reactive care towards care that embraces longitudinal, preventative, community-based and integrated approaches (12).

#### Expert panel eligibility, mandate and activities

Local and international experts ranging from GPs, specialists, academicians and health officers with documented experience in the field of MASLD and PC were eligible to join the multidisciplinary panel. Experts were identified by consortium members from local networks in Greece, Spain and the Netherlands, the European Society of Primary Care Gastroenterology (ESPCG) and other relevant scientific societies, including the European Association for the Study of the Liver (EASL). A minimum of 10 experts were expected to participate in the panel.

Experts provided scientific and clinical expertise to support the development of the MASLD algorithm and were invited to:

- Conduct an assessment of evidence base and needs related to MASLD screening, detection, and management;
- Support the creation of pathway objectives;
- Support the development in terms of clinical content and practical modalities;
- Review and provide feedback on the draft pathway synthesis;
- Provide consensus and final approval of the pathway;
- Overview adaptation of the pathway for use in the targeted countries.

#### Algorithm development procedures

Development activities followed the Plan-Do-Study-Act framework (13) and the pathway was designed as a standardized care algorithm where different tasks were defined, optimized and sequenced. It aimed to systematically identify and follow patients at risk for low-intermediate or high-level MASLD, beginning from PC and aiming to improve care quality and efficiency, professional coordination/cooperation and patient satisfaction. Using Continuous Quality Improvement elements (14), the algorithm’s framework, content and procedures were addressed, focusing on available guidelines and evidence regarding the use of serum markers, non-invasive and imagining techniques to assess advanced fibrosis. The expert panel consolidated local assessments and drafted the algorithm during the following phases:

##### Preparation phase

Local stakeholder meetings were held in each country prior to the international panel meetings to identify needs and priorities from each setting. An initial synthesis of algorithm elements and a guide to its implementation was produced based on individual country reports and a literature review. These were disseminated to experts before the panel meeting. The experts were asked to review the draft and suggest modifications via e-mail. Individual responses were collected and processed. Emerging questions were drafted and sent to experts for discussion in the main panel meeting.

##### Main phase

This included the meeting of the expert panel, which, due to COVID-19 restrictions, was held online (March 2022). During the meeting, experts were asked to reflect on the questions drafted in the preparation phase, which related to algorithm content and were organised into topics addressing:

1. Evidence-base, best-practices and pathway framework;
2. Model objectives and prioritization criteria;
3. Mapping the patient journey;
4. Clinical algorithm (decision nodes and process needing standardization).
5. Pathway implementation tools and supportive materials;
6. Assessment of risk level;
7. Patient education, behaviour change and self-management.

During the meeting the panel refined the draft pathway in terms of supporting background (evidence-base, best-practice criteria, and guidelines), implementation and evaluation. The meeting started with the agenda presentation and included small group discussions and plenary sessions moderated by a consortium member.

##### Consensus phase

Elements of the Rand/UCLA method (RAM) were used to reach consensus (15). The overarching themes, topics and conclusions produced by the expert panel meeting were summarized in a report that was circulated among all experts. Components identified by the expert panel meeting were then triangulated with information from other sources, including literature. All information was fitted into the pathway draft (algorithm and guide to implementation) which was finalized and approved by all experts through a final consensus.

##### Local adaptation phase

The pathway was developed in English and translated in Greek, Spanish and Dutch. Individual country meetings were held to address the potential necessity of further adaptations. Local GPs were also invited to comment on algorithm content, comprehensiveness and feasibility before evaluation.

#### Analysis/reporting

Description and outcomes of each process step were summarized in a final report.

### Pilot evaluation

#### Design

A controlled trial pilot study was conducted to evaluate the implementation of the proposed MASLD pathway compared to standard care. Eligible patients were assigned to either an ‘active’ or a ‘control’ GP practice. Patients were blinded to the type of practice they were assigned.

#### Setting and participants

This pilot was conducted in PC settings in Crete (Greece) and Barcelona (Spain). The pilot evaluation could not be conducted in the Netherlands due to logistic reasons, including inability of GPs to facilitate the study reporting post-COVID workload and limited MASLD interest. In each country, four GP practices served as study sites. As such, a total of eight GPs representing a range in gender, age, years of experience and area of practice were purposively selected to facilitate the study based on the following criteria:

1. Holder of specialty degree in GP and/or PC serving in public or private sector;
2. Service in a practice of a well-defined health area;
3. Minimum of 15 patients seen per day.

Patients consecutively visiting the selected GPs were considered eligible for participation based on the following criteria:

1. Metabolic dysfunction: presence of either overweight/obesity, type 2 diabetes, metabolic syndrome OR.
2. Hypertransaminasemia: raised ALT OR raised AST OR.
3. Confirmed MASLD: ultrasound or Fatty Liver Index (FLI) > 60 AND no other causes of liver disease AND no alcohol excess OR.
4. Presence of CVD: any diagnosis or on medication for CVD.

Eligibility criteria were assessed by research assistants through electronic/paper based medical records and based on the specific definitions provided in the pathway guide of Appendix 1. Patients unwilling or unable to provide signed informed consent and complete the procedures for any reason were excluded.

#### The intervention

Prior to study initiation, GPs caring for patients of the active group received training in pathway implementation and attended a MASLD eLearning developed by our research team and described elsewhere (16). GPs of the control group received no training and provided usual care. GPs of both groups were then allowed to perform their clinical practice as preferred. We hypothesized, however, that trained GPs would screen eligible patients for MASLD and would carry out the pathway procedures regarding referral and management of patients with risk of high-level MASLD in higher rates than GPs of the control group. According to pathway, screening included calculation of FIB-4 (next-to-patient; Appendix 1). Patients with FIB-4 < 1.30 were considered as having no sufficient evidence of liver fibrosis, thus not requiring referral. However, they were supported to modify their lifestyle and further managed in PC. For indeterminate FIB-4 (1.3 ≤ FIB-4 < 2.67), patients were referred for elastography and were further classified, with patients having risk of low-level MASLD (<7.9 kPa) retained for PC management. Patients with risk of high-level MASLD (FIB-4 ≥ 2.67 or elastography≥7.9 kPa) were directly linked to specialists.

#### Sampling and sample size

Patient sampling was consecutive from participating GP practices and not stratified. Rough sample size estimations, assuming that the number of the patients screened will be 4 times higher in the intervention group and that the number of patients diagnosed with advanced fibrosis will be 6 times higher in the intervention group than in the control (based on two-sided test, 80% power and alpha level of 0.05), suggested that 50 patients would need to be recruited per practice.

#### Study outcomes

Outcomes were assessed in both study groups at patient’s first visit in the practice (baseline) and at 4–8 weeks follow-up (September–December 2022). Primary outcomes were the number of patients screened, found with fibrosis, and referred to specialty care as measured at follow-up. Other variables assessed via patients’ self-report, medical records or physical examination, respectively, included demographic characteristics (age, gender, education), health habits (smoking, alcohol, diet), biomedical indexes (weight, height, blood pressure), existing and new laboratory tests (in particular for metabolic dysfunction and liver enzymes), existing and new diagnoses (particularly for liver diseases), existing and new medications, existing and new diagnostic tests (particularly FIB-4, elastography, liver ultrasound, liver biopsy).

#### Data collection tools and procedures

##### Baseline assessment

Data collection was parallel and same in the participating countries. In both study groups, research assistants assessed eligibility criteria for all patients consecutively visiting the GPs over a period of 2 weeks and invited them to participate using a detailed information sheet. Patients who provided signed informed consent completed the first part of a case report form (CRF), which was administered by research assistants and assessed sociodemographic characteristics and health habits. Using patients’ electronic medical records, research assistants also completed the second part of the CRF, which assessed medical history, including tests, examinations, diagnoses, and medications. Research assistants finally observed participants’ consultations with the study GPs and completed the third part of the CRF, assessing GPs’ practice regarding pathway implementation.

##### Follow-up assessment

Follow-up was performed 4–8 weeks after the baseline assessment in both study groups. Using patients’ medical records, research assistants completed the final part of the CRFs, which tracked patient outcomes, progress through the care system, and follow-up by recording referrals, decisive diagnoses, and new treatments.

#### Data analysis

Data were presented using descriptive statistics. Mann–Whitney U tests were performed to examine between-group differences in continuous variables. Fishers’ exact tests were performed in small samples to explore between-group differences in categorical variables, while *X*^2^ tests were used in larger samples. Statistical significance was set at *p* < 0.05 and analyses were performed using SPSS (Version 25.0. Armonk, NY: IBM Corp).

## Results

### Pathway development

#### Expert panel synthesis

The established expert panel included 10 international experts from three countries and multiple disciplines, namely general practice (*n* = 4), hepatology/gastroenterology (*n* = 2), public health (*n* = 2) and academia (*n* = 3). The panel exchanged several e-mail communications and conducted an expert meeting until consensus (March 2022).

#### Synthesis of the evidence base

The following clinical guidance and resources were used, among others, as the basis for expert panel discussions and pathway formation:

- EASL–EASD–EASO Clinical Practice Guidelines on the management of NAFLD (17);
- NICE. Non-alcoholic fatty liver disease (NAFLD) assessment and management (18);
- The Lancet Live Campaign (19);
- The Camden and Islington NAFLD pathway (9);
- Screening for NAFLD in PC (20, 21).

The following acknowledgments were also made by the expert panel before algorithm development, based on the synthesis of the evidence:

- PC is vital in preventing the development and progression of MASLD;
- Systematic response to abnormal liver blood tests and screening high-risk patients with referral to secondary care is necessary;
- A focus on managing metabolic comorbidities to reduce CVD risk and prevent MASLD complications is required;
- There is an unmet need for integrated interface between primary and secondary care with robust pathways for screening, fibrosis testing and subsequent referrals;
- Lack of such pathways results in missing a significant proportion of the risk population;

#### Pathway priority areas

Table 1 presents the priority topics and questions addressed by the expert panel during the preparation phase and based on which decisions on model content were taken. In summary these address the level of care to which the pathway should be implemented, expected implementation barriers, patient population to be screened by the pathway (including eligibility criteria), MASLD screening tools and MASLD management in PC.

**Table 1:** Questions addressed by the international expert panel on MASLD pathway priorities.

Who screens for MASLD? Which level of care? Primary or secondary care? What barriers are expected? Providers’ knowledge Availability of screening tools Costs Who/when to screen for MASLD? Which patients to target? Population screening or high risk only? When to screen? On regular visits or upon abnormal liver tests only? How to screen? Which tests to use? type of available tests risk classification thresholds patient preferences How to manage MASLD? What should MASLD management in primary care include? consolidate with evidence and previous work synthesize available guidelines reinforce doctor-patient communication

#### Final MASLD algorithm

In accordance with EASL recommendations and international evidence suggesting that almost 90% of unnecessary referrals for MASLD can be avoided by structured screening in PC, the expert panel consented that PC physicians are particularly suited to identify MASLD risk factors and determine respective risk of MASLD level (17, 20). Screening the general patient population was not considered as, of those, about 20–30% will have MASLD and 7–10% will develop complications. Instead, literature and EASL guidelines suggest that screening patients with risk factors, including obesity, type 2 diabetes and metabolic syndrome, is of particular importance, as over 75% of them will be identified with MASLD (17, 20). Taking into consideration the growing evidence on the association of MASLD with cardiovascular disease (CVD) morbidity and mortality, the expert panel included CVD diagnosis among the algorithm’s eligibility criteria for MASLD screening. In terms of screening tests to be employed by the algorithm, the decision was made based on availability in PC of partnering countries, with FIB-4 score and elastography primarily used for the detection of risk of fibrosis level. Apart from pharmacotherapy, focusing on lifestyle modification was deemed important based on literature (17, 21). Thus, the pathway further provided resources and guidelines for behavioural interventions, along with the specific MASLD training for PC providers that was developed by our research group and has been reported elsewhere (16).

As such, in a two-step clinical care pathway, patients with metabolic dysfunction, hypertransaminasemia, confirmed MASLD or cardiovascular disease are considered eligible for MASLD screening based on FIB-4. The algorithm classifies screened patients at risk of low-level (FIB-4 < 1.3), intermediate-level (1.3 ≤ FIB-4 < 2.67) or high-level MASLD (FIB-4 ≥ 2.67). Patients at risk of low-level MASLD are managed in PC, with the pathway providing the evidence-base, training resources and guidelines to support GPs perform behaviour/lifestyle modification interventions, treatment and follow-up. Patients with risk of high-level MASLD based on FIB-4 are directly referred to specialty care, with the pathway providing all the resources for care coordination and subsequent primary care monitoring. Patients with intermediate-level MASLD are referred for further examination with elastography and are subsequently classified as low-risk (<7.9 kPa or fibrosis stages F0/F1) or intermediate/high risk (≥7.9 kPa or fibrosis stages F2/F3/F4). Low-risk patients based on elastography are managed in primary care, whilst patients at intermediate/high risk are directly referred to specialty care with specific guidance on care coordination and subsequent primary care monitoring.

**Figure 1:**
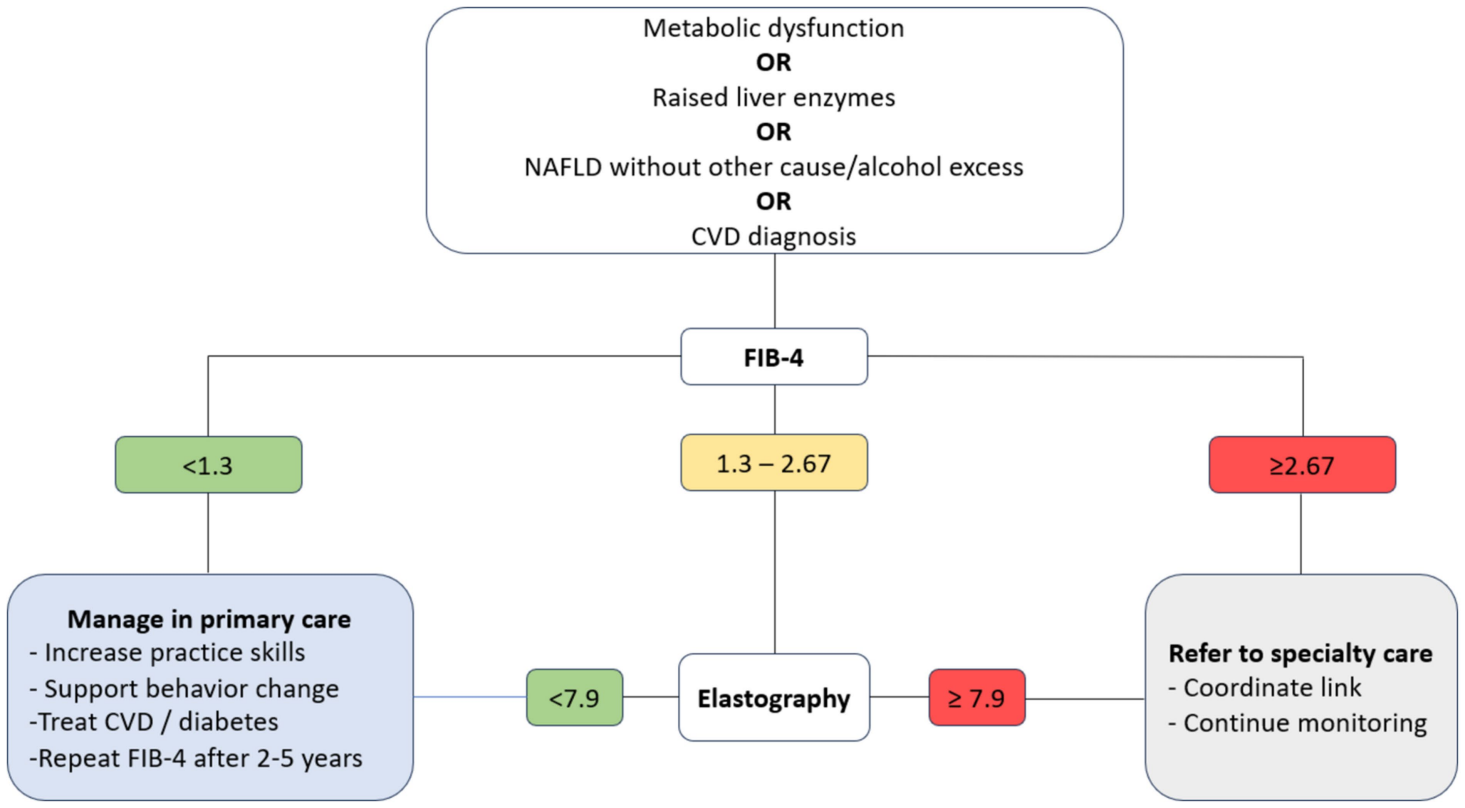
The final MASLD primary care pathway (short version).

The comprehensive version of the final clinical care pathway produced by the expert panel processes is illustrated in Figure 1, while its detailed version and associated implementation guide is provided in Appendix 1. All experts and all three countries endorsed the model without further adaptations apart from translation.

### Pilot evaluation

#### MASLD risk profile

In Spain, *N* = 37 participants were recruited at baseline (54.1% female, median age: 63 years). In terms of risk profile, 21.6% were smoking, 73.0% had BMI > 30, 37.8 and 45.9% had abnormal triglycerides and HDL respectively, 54.1% had increased fasting glucose, 83.8% had increased systolic blood pressure, while 56.8 and 62.2% had abnormal AST and ALT, respectively (Table 2).

**Table 2:** MASLD risk profile of *N* = 37 patients recruited at baseline in Barcelona, Spain.

Variable	*n*	%
Smoking (yes)	8	21.6
Body mass index		
25–29.9	9	24.3
≥30	27	73.0
Triglycerides (>150)	14	37.8
HDL (<50)	17	45.9
Fasting glucose (>100)	20	54.1
Systolic blood pressure (>130)	31	83.8
AST (9-32)	21	56.8
ALT (7-30)	23	62.2

In Greece, *N* = 182 patients were recruited at baseline (51.1% female, median age: 64 years). In terms of MASLD risk profile, 51.6% of Greek participants were found with metabolic dysfunction, 3.3% with hypertransaminasemia and confirmed NAFD respectively, while 68.1% had a confirmed CVD diagnosis (data not shown).

#### Existing tests and diagnoses

In Spain (Table 3), a confirmed MASLD diagnosis was found in the records of 18 patients (48.6%). Twenty-one patients (56.6%) already had a FIB-4 score, with 13 (61.9%) of them classified as having risk of low-level MASLD and 8 (38.1%) as having risk of intermediate-level MASLD. An ultrasound examination was present for 12 (32.4%) patients, indicating hepatic steatosis for 10 (83.3%) of them. Nine (24.3%) patients had an existing elastography, with eight (88.9%) identified at low risk and one (2.7%) at high risk. Finally, two (5.4%) patients had a liver biopsy in their records.

**Table 3:** Existing MASLD assessments and diagnoses for *N* = 37 patients in Barcelona, Spain.

Variable	*n*	%
Alcoholic Liver Disease	2	5.4
MASLD	18	48.6
MASH	2	5.4
Fibrosis	1 (stage 4)	2.7
Type 2 diabetes	20	54.1
CVD	29	78.4
Anti-HCV, HBsAg, anti-HBc	5	13.5
Positive	0	0
FIB-4	21	56.8
Risk of low-level MASLD	13	61.9
Risk of intermediate-level MASLD	8	38.1
Risk of high-level MASLD	0	0
Ultrasound	12	32.4
Hepatic steatosis	10	83.3
Elastography	9	24.3
Low-risk	8	88.9
Intermediate-/high-risk	1	11.1
Biopsy	2	5.4

In Greece, no confirmed MASLD diagnoses were found in patients’ records. However, two (1.5%) diagnoses of alcoholic liver disease (ALD) were documented (ALD was not part of MASLD at the time when these diagnoses were recorded). None of the patients had ever had a FIB-4 score, an elastography or a liver biopsy recorded. Still, an ultrasound result was available for 138 (75.8%) patients, indicating MASLD for 71.4% of them (data not shown).

#### Pathway implementation: screening

As presented in Table 4, in Spain, patients in the active group (*n* = 17) received a FIB-4 score more frequently than patients in the control group (*n* = 20) and this difference was statistically significant (*n* = 16 or 94.1% vs. *n* = 10 or 50.0%, *p* = 0.004). From patients having risk of intermediate-level MASLD based on FIB-4 (*n* = 1 or 31.3% active vs. *n* = 4 or 20.0% control), one (5.9%) and three (15.0%) were referred for elastography in the active and control group, respectively, (*p* = 0.609). One-month follow-up data suggest that, from the four elastographies ordered in total, only one had been performed within the study time frame. This concerned a control patient and indicated a low risk of fibrosis (5.2 kPa, results not shown).

**Table 4:** Primary care MASLD screening based on pathway implementation for *N* = 37 patients in Barcelona, Spain.

Variable	Active group (*n* = 17)	Control group (*n* = 20)	*p*-value
FIB-4 performed	16 (94.1%)	10 (50.0%)	0.004
Risk of low-level MASLD	11 (68.8%)	6 (60.0%)	
Risk of intermediate-level	5 (31.3%)	4 (20.0%)	
Risk of high-level MASLD	0 (0%)	0 (0%)	
Elastography referral	1 (5.9%)	3 (15.0%)	0.609

In Greece, no FIB-4 scores were performed and no elastographies were ordered by GPs of either the active or the control group.

#### Pathway implementation: management

As shown in Table 5, in terms of PC management, GPs in the active group of Spain intervened significantly more frequently compared to the control group in terms of weight loss (70.6% vs. 10.0%, *p* < 0.001), alcohol regulation (52.9% vs. 0%, *p* < 0.001) and smoking cessation (29.4% vs. 0%, *p* = 0.005). They also communicated a MASLD diagnosis at higher rates (88.9% vs. 30.0%) and kept patients in PC for monitoring and management (17.6% vs. 20.0%), however these differences were not statistically significant. One-month follow-up data suggest that the FIB-4 score was repeated within the study time frame for two control group patients (results not shown).

**Table 5:** Primary care MASLD management based on pathway implementation for *N* = 37 patients in Barcelona, Spain.

	Active group (*n* = 17)	Control group (*n* = 20)	*p*-value
Weight loss			
Recommended	4 (23.5%)	16 (80.0%)	<0.001
Intervened	12 (70.6%)	2 (10.0%)	
None	1 (5.9%)	2 (10.0%)	
Alcohol regulation			
Recommended	1 (5.9%)	6 (30.0%)	<0.001
Intervened	9 (52.9%)	0 (0%)	
None	7 (41.2%)	14 (70%)	
Smoking cessation			
Recommended	0 (0%)	4 (20.0%)	0.005
Intervened	5 (29.4%)	0 (0%)	
None	0 (0%)	0 (0%)	
Treatment			
Prescription	9 (52.9)	6 (30.0%)	0.193
No prescription	8 (47.1%)	14 (70.0%)	
Diagnosis communicated	9 (52.9%)	10 (50.0%)	0.858
MASLD	8 (88.9%)	3 (30.0%)	
Metabolic disorder	1 (11.1%)	2 (20.0%)	
Other	0 (0%)	5 (50.0%)	
Follow-up arranged			
Yes	17 (100%)	20 (100%)	-
No	0 (0%)	0 (0%)	
Referral performed	3 (17.6%)	4 (20.0%)	0.855
Hepatologist	3 (100%)	2 (50%)	

## Discussion

### Summary of findings

This study provides insights on the development and pilot implementation of a MASLD clinical care pathway for use in PC of three European countries. Pathway development was based on expert opinion, while its pilot evaluation was conducted in a controlled study in Spain and Greece. In Spain, despite the small study sizes, GPs exposed to the MASLD pathway screened significantly higher proportions of patients using the FIB-4 score compared to GPs who followed usual care procedures. Given that our algorithm provided a detailed framework with explicit guidance and resources for MASLD management in PC (including tools for behavioural change interventions), exposed GPs indeed documented significantly higher rates of performance of such interventions, compared to GPs of the control group. Contrary to Spain, no implementation of the MASLD was observed among both exposed and not exposed GPs, which is indicative of local context particularities and warrants further investigation. Logistic issues precluded evaluation in the Netherlands. This variability in implementation success across participating countries is indicative of the challenges related to tailoring and integrating such pathways in diverse and complex clinical systems across Europe and warrants further investigation.

### Comparison with literature

Despite large differences in study designs, our findings align with published studies assessing the effectiveness of clinical care algorithms for MASLD management in PC. A prospective study from the UK examining the implementation of a similar pathway among PC patients with screening based on FIB-4, suggested significant improvements in the detection of advanced fibrosis and cirrhosis, while reducing unnecessary referrals in patients with MASLD, highlighting the importance of such strategies for improving resource use and patient outcomes (9). In another study estimating the proportion of patients with type 2 diabetes that should be referred to hepatologists, it was found that the use of age-adjusted FIB-4 cut-offs can lead to more sustainable referrals to specialists (22). Similarly, a study assessing the diagnostic performance of nine clinical non-invasive fibrosis models in MASLD, indicated that the combination of these models performed best for diagnosing advanced fibrosis, providing valuable reference tools for clinical practice (23). Finally, several other studies and individual actions provide algorithms to support PC professionals screen patients with MASLD using liver enzymes, assess advanced fibrosis using prediction rules and determine when to refer patients to specialists (21, 24).

The results observed for Greece are indicative of the context within which the study was performed. The prevalence of MASLD in Greece is largely unknown, however, it is estimated that it exceeds 30% of the general population (25). Moreover, evidence suggests that MASLD is increasing in parallel with risk factors including obesity and diabetes (26, 27). Despite this growing burden, previous work of our group shows that factors driving health behaviour, such as MASLD health literacy and illness perception, are limited among Greek PC patients (28). At the same time, a recently published report of our group, also highlights the low levels of MASLD-related knowledge, confidence and clinical practices among Greek GPs, which however present statistically significant increases after exposure to a newly developed professional training intervention (16). As such, it is not surprising to observe these low levels of pathway implementation and the absence of differences between the active and control GP groups of this study.

### Strengths and limitations

To our knowledge, this is the first study that mobilizes the expertise of an international multidisciplinary panel in an attempt to develop and evaluate an integrated clinical care pathway for MASLD screening, diagnosis and referral in Greek, Spanish and Dutch PC. It is also among the few that provides model implementation data on the outcomes of a PC algorithm for MASLD using FIB-4 for risk stratification in Europe. Although there has been some discussion about the accuracy of FIB-4 and its value for the comprehensive management of MASLD patients considering the complexity of the disease (29), it is a practical tool suggested by international clinical practice guidelines (17) and, often the only available option in certain PC settings, like Greece.

However, our study has several limitations. First, the small sample sizes at both the GP and the patient levels, together with the lack of robust sample size estimation and proper statistical power calculation, do not allow for robust conclusions and generalizability of the results. Moreover, the design of this study precludes assessment of the prospective and long-term impact of our pathway to properly determine its effectiveness. Although an external research assistant conducted the data collection in most cases, it is possible that GPs of the active group may have been more motivated to implement the clinical pathway due to their exposure to the training. Finally, the particularities of each study setting and the variability in implementation success across participating settings must be taken into account when making cross-country comparisons and interpreting overall results.

### Implications for research, policy and practice

This study was the pre-final part of a larger international collaborative project on MASLD/MASH models in primary care.^1^ According to the highlights of the EASL liver commission (30), in a model of care process, this project compiled straightforward algorithms for MASLD screening and referral, new modes of collaborative care and explicit tools for PC management, including behavioural interventions, with the goal of achieving meaningful changes in clinical practice standards. Countries in southern Europe generally lack such multidisciplinary partnerships in PC, while a focus on early disease identification and management of risk factors is not regularly part of clinical practice priorities (31, 32).

Particularly for MASLD, despite the availability of practice guidelines on its clinical management, including the joint guidance from EASL, EASD and EASO, in many healthcare settings no pathways exist or, if they do, they are frequently empirical and not evidence-based (17). Furthermore, under systems’ fragmentation and lack of integration and coordination, insufficient services are provided to patients along the MASLD continuum, negatively impacting patient outcomes (33). To improve care for patients with MASLD, it is necessary for health policies and strategies to build on multidisciplinary, context-driven, patient-centred frameworks that provide explicit guidance on MASLD care, an action that has been proven effective in improving care for other diseases (34). Aiming to contribute to bridging the gap between guidance and practice and address the increasing need for best-practice care for patients with MASLD, our pathway assets, along with existing evidence and expert recommendations (35), can be used by stakeholders in the development of high-level models of care to improve the future management of this condition.

Further prospective and longitudinal research is required to confirm the (cost)effectiveness of our proposed PC pathway and the best methods to further screen for advanced fibrosis. In particular, it is imperative that subsequent studies address the limitations through larger, more diverse study populations and methodologies that allow for a comprehensive assessment of the algorithm’s effectiveness, sustainability, and adaptability across different healthcare contexts. However, with the growing burden of MASLD as a global public health issue, primary care has an important role to play in terms of screening patients and preventing the development and progression of MASLD (36). Along with building robust pathways to support the interface between primary and secondary care, raising public and professional MASLD awareness and education and increasing skills on the active management of cardiovascular risk factors can result in better identification of high-risk patients who will benefit the most from early intervention (37). Given the ongoing PC reforms in settings like Greece, with positive results that include the establishment of community-based multidisciplinary health teams (38), the time to act for MASLD is now.

## Conclusion

This study points to better performance in MASLD screening and management for GPs exposed to a MASLD PC pathway compared to GPs attending routine practice, although further research is required to overcome limitations and confirm results. Cross-country variations indicate the different levels of preparedness for MASLD actions and highlight the need for context-driven approaches to increase MASLD screening, management and referral among all settings. Prospective and longitudinal studies are necessary to assess the long-term effects of our pathway and determine its potential for scaling up and integration.

## Data Availability

The raw data supporting the conclusions of this article will be made available by the authors, without undue reservation.

## Footnotes

^1^ http://www.nash.med.uoc.gr/
