# Supplementary material for: Development and pilot evaluation of an evidence-based algorithm for MASLD (formerly NAFLD) management in primary care in Europe
Source: Front Med (Lausanne). 2024 Nov 21;11:1383112. doi: 10.3389/fmed.2024.1383112 (PMC11617198; doi:10.3389/fmed.2024.1383112)
Supplement: Supplementary file 1 [file Data_Sheet_1.docx]

# **Appendix 1: Detailed MASLD algorithm and implementation guide**

**INTRODUCTION**

*This primary care pathway was co-developed by an international multidisciplinary panel of primary and specialty care professionals, public health experts and academicians. It is intended to provide a simple approach for use in the busy primary care setting to facilitate cross-departmental collaboration and shared care paths. Primary care pathways for MASLD/MASH can contribute to the timely disease identification and provide evidence-based support to physicians and their patients, while improving appropriate referral to specialty care.*

Metabolic dysfunction-associated steatotic liver disease (MASLD, formerly NAFLD) represents a spectrum of disease that ranges from simple fatty liver (steatosis) to fatty liver with cell inflammation and ballooning (Metabolic dysfunction-associated steatohepatitis - MASH), which can progress to liver fibrosis and ultimately cirrhosis, liver failure, hepatocellular carcinoma and death. Advanced fibrosis due to MASH represents a significant unmet medical need, for which there are limited therapies, non-invasive screening, diagnostic and monitoring tools, and poorly defined models of patient care.

Patients with advanced fibrosis commonly have comorbid metabolic conditions, including obesity, type 2 diabetes mellitus (T2DM) and dyslipidaemia, which are primarily managed in primary care. Many of these conditions are key predictors for the development and progression of fibrosis due to MASH. Consequently, patients with these comorbidities represent a high-risk population, potentially suitable for MASLD/MASH screening.

Appropriate suspicion, screening, identification and linkage to care of patients with advanced fibrosis remain a significant unmet need. Key success factors for identification of MASLD patients and subsequent care delivery includes use of simple non-invasive tests, standardized referral and treatment algorithms, and multi-disciplinary collaboration between primary and specialty care.

In the absence of comprehensive guidance and availability, however, use of non-invasive tests and proper referral to specialty care for high-risk patients is challenging for primary care physicians. Patients with mild disease are often referred when the appropriate preventative interventions of lifestyle changes can be delivered effectively in primary care. In contrast, advanced fibrosis or cirrhosis is often under-estimated, remaining undetected and leading to late diagnosis of progressed disease.

The proposed primary care pathway for MASLD aligns available evidence and best practices to:

- Provide a simplified approach to identify patients at high-risk for advanced fibrosis, employing currently available and approved non-invasive tests
- Establish referral and treatment pathways for high-risk patients, facilitating cross-departmental collaboration
- Use implementation science to evaluate screening for MASLD/MASH in primary care settings and subsequent linkage to specialty care

**DETAILED ALGORITHM FOR MASLD MANAGEMENT IN PRIMARY CARE**

| **A. DETERMINE ELIGIBILITY (people “at-risk”)**   1. **Metabolic dysfunction**: overweight/obesity^1^ **OR** type 2 diabetes **OR** MetS^2^   **OR**   1. **Liver dysfunction:** raised ALT^3^ **OR** raised AST^4^   **OR**   1. **MASLD**: Ultrasound or FLI>60^5^ **AND** no other causes of liver disease^6^ **AND** no alcohol excess^7^   **OR**   1. **CVD**^8^: any diagnosis or on medication for CVD | | | | | | |
| --- | --- | --- | --- | --- | --- | --- |
|  |  |  |  |  |  |  |
|  |  |  |  |  |  |  |
|  |  |  |  |  |  |  |
|  |  |  | **B. PERFORM**  **FIB – 4**^9^ |  |  |  |
|  |  |  |  |  |  |  |
|  |  |  |  |  |  |  |
| **Low Risk**  FIB-4<1.3 |  |  | **Intermediate Risk**  1.3≤FIB-4<2.67 |  |  | **High Risk**  FIB-4≥2.67 |
|  |  |  |  |  |  |  |
|  |  |  |  |  |  |  |
|  |  |  | **C. ORDER/PERFORM**  **ELASTOGRAPHY OR ELF**^10^ |  |  |  |
|  |  |  |  |  |  |  |
|  |  |  |  |  |  |  |
|  |  | **Low risk**  <7.9kPa  (stages F0/F1) |  | **Intermediate/**  **High** **risk**  ≥7.9kPa  (stages F2/F3/F4) |  |  |
|  |  |  |  |  |  |  |
|  |  |  |  |  |  | **E. COORDINATE REFERAL AND PRIMARY CARE MONITORING** |
| **D. MANAGE IN PRIMARY CARE**^11^  1. Increase practice skills  **2. Behaviour/lifestyle change**: weight, smoking, alcohol  3. **Treatment**: CVD and diabetes  4. **Repeat FIB-4:** after 2-5 years, based on risk and MASLD duration  **5. Follow-up:** periodic examination, annual LFTs (if necessary) |  |  |  |  |  |  |

**QUICK GUIDE TO MASLD ALGORITHM IMPLEMENTATION**

The proposed pathway is a simple, three-step algorithm for MASLD identification in primary care, starting with patient screening for basic MASLD risk factors and proceeding with the use of non-invasive tests (FIB-4 and transient elastography) to identify patients at risk of advanced fibrosis. The algorithm also provides practical guidance and tools on how to manage low-risk patients in primary care and further determines referral paths for intermediate- and high-risk patients.

In summary, the main steps of the proposed pathway are (please refer to the subsequent section for specific details):

1. Screen for patients at-risk during daily practice including:
   1. People with **MASLD -related risk factors** (i.e. overweight/obesity, type 2 diabetes, metabolic syndrome, hyperlipidaemia)
   2. People with **abnormal levels of liver enzymes** (incidental or purposive finding)
   3. **People with MASLD**, having ruled out other causes of liver disease (including excessive alcohol intake). If other causes are identified, treat or refer to specialist.
2. Perform **FIB-4** to patients at-risk identified in step 1.
   1. Manage low-risk patients (FIB-4<1.3) in primary care
   2. Refer high-risk patients (FIB-4≥2.67) to specialist and coordinate shared care management.
3. Perform (if available in primary care) or order **transient elastography** to intermediate-risk patients (1.3≤FIB-4<2.67) identified in step 2.
   1. Manage low-risk patients in primary care (fibrosis stages F0 and F1)
   2. Refer intermediate- and high-risk patients (stages F2, F3, F4) to specialist and coordinate shared care management.

**EXPANDED DETAILS**

1. **Overweight**: waist circumference between 94-102cm in men and 80-88cm in women

**Obesity**: waist circumference >102cm in men and >88cm in women

1. **MetS**: Metabolic syndrome, identified based on NCEP ATP III definition if three or more of the following risk factors were present:
   1. Abdominal obesity: waist circumference: ≥102 cm for males and ≥88 cm for females.
   2. Dyslipidaemia: triglycerides: ≥150 mg/dL or on drug treatment for elevated triglycerides.
   3. Dyslipidaemia: HDL cholesterol: <40 mg/dL for males and <50 mg/dL for females or on drug treatment for reduced HDL cholesterol.
   4. Hypertension: systolic blood pressure: ≥130 mmHg or diastolic blood pressure ≥85 mmHg or on antihypertensive drug treatment in a patient with a history of hypertension.
   5. Hyperglycaemia: fasting glucose: ≥100 mg/dL or on drug treatment for elevated glucose.
2. **ALT**: alanine transaminase. Indicatory abnormal levels (also depending on lab):
   - 1. Men: 10-55 IU/L
     2. Women : 7-30 IU/L
3. **AST**: aspartate transaminase. Indicatory abnormal levels (also depending on lab):
   - 1. Men : 10-40 IU/L
     2. Women : 9-32 IU/L
4. **Fatty Liver Index (FLI) calculator**: <https://www.mdcalc.com/calc/10001/fatty-liver-index>
5. **Other causes of liver disease**:
   - 1. **Liver steatosis**: differential diagnosis
     2. **Drug induced steatosis**:
        1. Corticosteroids
        2. Tamoxifen
        3. Methotrexate
        4. Antiretroviral therapy
     3. **Nutrition**:
        1. Rapid weight loss
        2. Parenteral nutrition
        3. Anorexia
     4. **Alcoholic steatosis**
     5. **Rare diseases**
        1. Lipodystrophy
        2. Hypothyroidy
        3. Wilson disease
        4. Hemochromatosis
6. **Excessive alcohol intake**:
   - 1. Men: > 4 drinks on any day or > 14 drinks per week
     2. Women: > 3 drinks on any day or > 7 drinks per week
7. **CVD**: cardiovascular diseases including – but not limited to- atherosclerosis, stroke, coronary heart disease, ischaemic heart disease, heart failure.
8. **FIB-4 calculator**: <https://www.mdcalc.com/calc/2200/fibrosis-4-fib-4-index-liver-fibrosis>
9. **Elastography or ELF** depending on what is available in local primary health care (PHC)
10. **Clinical tools for MASLD management in primary care**:

| **Domain** | **Action** | **Tools** |
| --- | --- | --- |
| **1. Increase practice skills** | Train in MASLD management in primary care | eLearning course for NAFLD in primary care: <https://primary-health-training-hub.com/nafld-2/> |
|  | Follow clinical practice guidelines | - EASL-EASD-EASO Clinical Practice Guidelines:   <https://www.journal-of-hepatology.eu/article/S0168-8278(15)00734-5/fulltext>   - AASLD Clinical Practice Guidance:   <https://www.aasld.org/practice-guidelines/diagnosis-and-management-non-alcoholic-fatty-liver-disease> |
| **2. Behaviour and lifestyle change** | Monitor weight loss | - Calculate Body Mass Index (BMI) and measure waist circumference in every visit - Use Very Brief Advice (VBA) and Motivational Interviewing (MI) techniques - Refer to dietologist |
|  | Support smoking cessation | - eLearning and clinical tools to support smoking cessation in primary care: <http://titangc.uoc.gr/en.html> - eLearning and tools to reduce passive smoking: <https://primary-health-training-hub.com/secondhand-smoke/> |
|  | Monitor alcohol intake | - Assess intake in every visit: AUDIT-C questionnaire: - <https://www.mdcalc.com/calc/2021/audit-c-alcohol-use> - <https://cde.drugabuse.gov/instrument/f229c68a-67ce-9a58-e040-bb89ad432be4>) - Use VBA and MI - Refer to specialized quit services |
| **3. Treatment** | CVD | EPCCS Guidelines for primary care: <https://ipccs.org/epccs-guidance-documents/> |
|  | Type 2 diabetes | - EASD Guidelines for primary care: <https://www.escardio.org/Guidelines/Clinical-Practice-Guidelines/Diabetes-Pre-Diabetes-and-Cardiovascular-Diseases-developed-with-the-EASD> - PCDE Guidelines for medical treatment in primary care: <https://www.guidelines.co.uk/diabetes/pcde-pharmacological-management-of-type-2-diabetes/455520.article> |
| **4. Repeat FIB-4** | FIB-4 calculation | <https://www.mdcalc.com/calc/2200/fibrosis-4-fib-4-index-liver-fibrosis> |
| **5. Follow-up and care coordination** | Referral to hepatologist | - Elastography - Enhanced Liver Fibrosis (ELF) test |
|  | Communication with laboratory personnel | - Liver function tests (LFT) including albumin, total protein, ALP, ALT, AST, bilirubin - Other lab tests |
